# An Overview of 3D Bioprinting Impact on Cell Viability: From Damage Assessment to Protection Solutions

**DOI:** 10.3390/jfb16120436

**Published:** 2025-11-25

**Authors:** Sara Manzoli, Elena Merotto, Martina Piccoli, Pierangelo Gobbo, Silvia Todros, Piero G. Pavan

**Affiliations:** 1 Department of Industrial Engineering, University of Padova, Via Venezia 1, 35131 Padova, Italy; sara.manzoli@unipd.it (S.M.); elena.merotto@phd.unipd.it (E.M.); piero.pavan@unipd.it (P.G.P.); 2 Fondazione Istituto di Ricerca Pediatrica Città Della Speranza, Corso Stati Uniti, 4 F, 35127 Padova, Italy; martina.piccoli@aulss8.veneto.it; 3 Advanced Cellular Therapy Laboratory, Aulss 8 Berica, Vicenza Hospital, Contrà S. Francesco 41, 36100 Vicenza, Italy; 4 Department of Chemical and Pharmaceutical Sciences, University of Trieste, Via L. Giorgieri 1, 34127 Trieste, Italy; pierangelo.gobbo@units.it; 5 National Interuniversity Consortium of Materials Science and Technology, Unit of Trieste, Via G. Giusti 9, 50121 Firenze, Italy

**Keywords:** bioprinting, shear stress, cell viability, bioink formulation

## Abstract

Three-dimensional (3D) bioprinting has become a widely exploited tissue engineering technique for producing functional constructs that can mimic and replace native tissues. To this end, different printing strategies can be adopted, including inkjet-based, light-assisted, and extrusion-based bioprinting. Despite the great improvements that these innovative techniques introduce, cell viability maintenance during and after the bioprinting process remains a challenging open question. Indeed, the reduction in cell viability is generally related to several crucial conditions during printing, such as high shear stresses and a nutrient-deficient environment of printed constructs. In this work, the current literature on 3D bioprinting technologies is reviewed, focusing on the level of cell damage that can be imparted during biomaterial printing. In particular, extrusion bioprinting, extrusion-associated shear stress and its impact on cell viability are described in detail. The simulation of the bioprinting process through computational fluid dynamics is proposed as an appropriate method to analyze the parameters involved during bioprinting. Moreover, the viability of cells encapsulated into bioink is discussed, as well as literature techniques aimed at enhancing it by both biomaterial modifications and cell micro-encapsulation.

## 1. Introduction

In recent years, a major clinical issue is represented by the disparity between the demand for organ transplantation and their actual availability. It is in this context that the tissue engineering (TE) approach started to move its first steps and provided radical solutions to existing clinical problems [1,2,3,4,5,6,7]. TE approaches indeed possess relevant clinical advantages that offer customized biocompatible solutions to patient-specific needs, promoting the human body’s natural healing capacities through precisely modified bioengineered scaffolds [8]. Despite these advantages, only some types of organs or tissues, such as skin, bladder, and blood vessels, have been produced so far, due to their simpler microstructures [9,10]. In case of more complex organs and tissues, conventional TE techniques face some limitations, and only improved methods of biofabrication enable the development of complicated and hierarchically organized tissues. Indeed, biofabrication techniques allow for spatial control over the construct being produced, since cellular and non-cellular materials can be arranged in space, mimicking complex tissue structures [6,9,11].

Biofabrication techniques [12] include 3D and 4D printing, light-based technologies such as stereolithography and two photon polymerization, fused deposition modeling, bioplotting, wet spun automated extrusion systems, ink-jet bioprinting, and electrospinning. These methods allow for spatial control over cellular and non-cellular materials, mimicking complex tissue structures.

Among these techniques, 3D bioprinting has gained widespread use due to its precision, reliability, and scalability. It allows for the simultaneous deposition of biomaterials and cells, creating accurate microenvironments with resolution down to micrometers [10,13,14,15,16]. Unlike other TE approaches that involve cell seeding on scaffolds, 3D bioprinting allows for simultaneous deposition of biomaterials and cells, leading to cell-appropriate localization into optimized microenvironments [6]. Moreover, it offers control over scaffold properties, such as porosity, size, and shape, and internal architecture [6,17].

**Figure 1:**
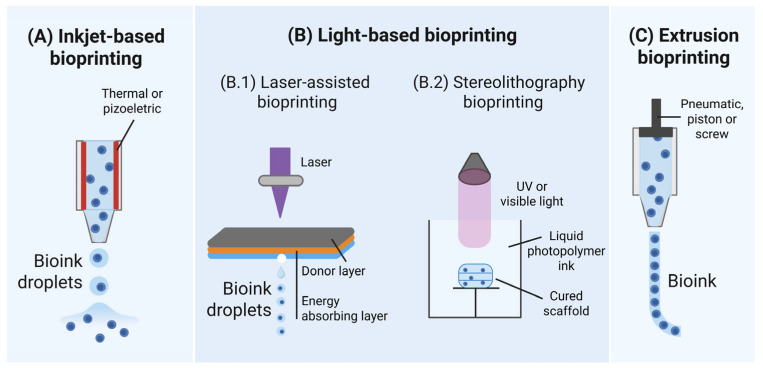
Schematic representation of different bioprinting approaches. (**A**) Inkjet bioprinting involves the deposition and collection of small bioink droplets onto a collecting plate; (**B**) Light-based bioprinting: (**B.1**) Laser-assisted bioprinting exploits a laser source to rapidly heat a donor material layer (gray), which subsequently forms a bubble into the bioink layer (blue); (**B.2**) Stereolithography bioprinting uses UV and visible light to selectively crosslink the bioink with a layer-by-layer motif; (**C**) Extrusion bioprinting typically involves pneumatic/piston or screw driven bioink ejection from a material tank. Created in BioRender.

Three-dimensional bioprinting generally comprises three main methods: inkjet-based, light-assisted, and extrusion-based bioprinting (Figure 1) [3,6,18]. Each of these methods has its own strengths, drawbacks, and limitations concerning cell viability. Therefore, selecting the appropriate processing protocol is crucial, considering the specific goals and application.

In this review, after a brief description of the major applications and 3D bioprinting technologies, the side effects of the bioprinting process will be described, focusing on cell-associated injury and suffering [19]. Thus, cell viability consequent to bioprinting will be analyzed together with the causes of its reduction, with a special focus on extrusion-based printing. The involvement of printing parameters is described: nozzle geometries and shear forces associated with printing will be evaluated.

To further predict the effect that the printing process exerts on cell viability, computational fluid dynamic (CFD) models will be introduced: cell-associated shear stresses will be quantified, and the cell damage ratio will be introduced as a viability indicator. Finally, literature studies aiming at improving cell viability through biomaterial modification and cell micro-encapsulation will be presented.

## 2. Bioprinting Technologies and Associated Cell Injuries

Conventional TE manufacturing techniques involve the production of a synthetic or biological scaffold over which cells are typically seeded and let grow and proliferate. Three-dimensional bioprinting started to become more and more explored once the need to produce complex morphological and functional structures was introduced. Indeed, 3D bioprinting technologies can lead to a precise deposition of cells and biomaterials, following a precise 3D spatial scheme that mimics the complex microstructural organization of tissues. Biomimicry, autonomous self-assembly, and tissue-building block production are the keywords associated with 3D bioprinting [7,20]. Among the most exploited bioprinting techniques, inkjet, light-based, and extrusion have to be mentioned. Even if these methods are associated with the ability to produce very complex structures, they are also accompanied by non-negligible problems, such as cell damage and cell viability threats during and after bioprinting, as discussed in detail in the following sections.

### 2.1. Inkjet Bioprinting

Inkjet bioprinting is a widely employed high-speed, low-cost technique with high resolution (up to 50 μm) and reliable bioink deposition control [3,20,21,22,23,24]. However, it has limitations in material range (viscosity 3–30 mPa·s) and maximum cell concentration (about 10^6^ cells/mL) [18,25]. It uses thermal or piezoelectric energy to convert biomaterials into ink droplets [26], with precise cell positioning up to obtaining single cell-based droplets [27]. This technique requires biomolecules and living cells suspended in biomaterials with controlled viscosity (3.5–12 mPa·s) [21,28].

Major concerns include clogged pores, bioink droplet dimensional changes, thermomechanical shear stress, and potential cell damage [29]. Thermal inkjet printing can reach high temperatures (up to 300 °C) that can lead to cell damage, although bioink temperature typically increases only 4–10 °C [24,30]. Low cell densities are preferred due to reduced shear stress at the nozzle clogging, but impact cell viability and tissue formation. Droplet drying [28] and unreliable cell encapsulation due to low concentrations of the bioink [20,22] are additional challenges.

In electrostatic drop-on-demand (DOD) inkjet printing, the bioink pressure change is induced by the deflection of a pressure plate. Generally, DOD techniques need high-pressure parameters, which negatively affect cell-laden bioinks and cause cell membrane damage [28]. Continuous inkjet (CIJ) printing exploits the Rayleigh-Plateau principle to generate a continuous stream of droplets [28,31]. However, CIJ is rarely used for biological constructs, due to the need for electrically conductive fluids and a high contamination risk [32].

Conversely, electrohydrodynamic jet bioprinting applies an electric field to deposit bioink droplets, avoiding high mechanical pressure, which reduces immediate cell damage. This process deforms the bioink into a cone (i.e., Taylor cone) for deposition. Nonetheless, the high voltage may cause long-term cell viability problems [1,28,33].

Regardless of the printing technique applied, shear stress is an inevitable and critical phenomenon for TE [34], influenced by nozzle diameter, printing pressure, and viscosity of the dispensing medium. Indeed, maintaining cell viability is essential for obtaining functional scaffolds.

#### Cell Damage in Inkjet Bioprinting

**Figure 2:**
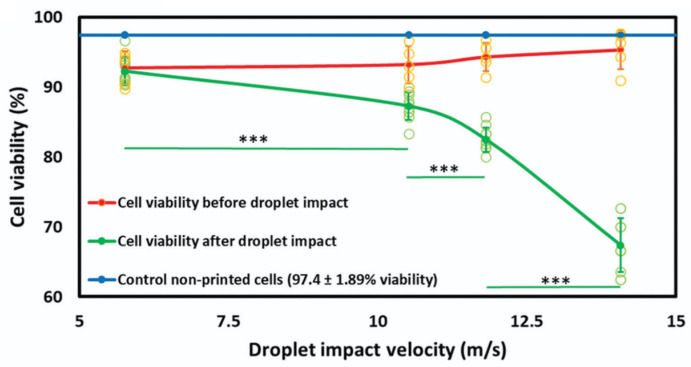
Effect of droplet impact velocity on printed cell viability before and after hitting the substrate surface. Reprinted from [36] and licensed under the Creative Commons Attribution 4.0 International license. (***) indicate significantly different values with *p* < 0.001.

Shear stress during inkjet printing mainly occurs when bioink droplets are ejected. Its magnitude is directly related to the bioink’s viscosity and shear rate. Stress intensity varies across the droplet, being higher in the lower region; generally, smaller droplets inflict less cellular damage. Cells closer to the nozzle walls experience deadlier shear stress compared to those near the central axis [28,35]. Cell damage is also correlated with shear stress exerted at the nozzle walls: indeed, during bioink ejection, deadly cells are localized near the nozzle walls, while viable cells are found near the nozzle axis. One proposed method to reduce shear stresses involves enlarging the nozzle valve [28,34]. However, this leads to larger droplets and fluid flow, compromising both printing resolution and shape fidelity. Shear stress is also exerted during the droplets landing, with droplet impact velocity and droplet volume (Figure 2) critically affecting the viability [36]. This issue can be overcome using higher cell concentrations that decrease impact velocity, thus enhancing cell survival and reducing droplet dispersion. Otherwise, this effect can be mitigated by using appropriate landing substrate coatings, often a selected hydrogel enriched with cell adhesion and growth factors [21].

Other critical factors affecting cell viability are the evaporation process after droplet casting and the operating temperature of the nozzle [19,37,38,39]. During thermal actuation, the ink reservoir temperature can exceed 300 °C. Higher magnitudes of these conditions increase the probability of cell damage, suggesting that minimizing the printing process timescale can improve viability [28].

It has been successfully used to create “lung-on-a-chip” models for drug testing [40]. Furthermore, thermally bioprinted scaffolds of alginate and gelatin, containing human microvascular endothelial cells, have been shown to augment native vasculature in animal models. This occurs because the jetted cells are stimulated to produce pro-angiogenic factors [41].

### 2.2. Light-Based Bioprinting

Light-based bioprinting exploits laser energy to precisely arrange a cell-laden bioink in a 3D arrangement [42]. The two most common techniques in this category are: laser bioprinting and stereolithography (SLA). Laser bioprinting has received attention for its ability to produce high-resolution scaffolds associated with high cell viability. As a nozzle-free method, it avoids clogging and allows the use of highly viscous bioinks (1 ÷ 300 mPa·s) [13,21,43].

The mechanism (known as Laser-Guided Direct Writing) involves a laser beam (700–1000 nm) that pushes cells onto the substrate by exploiting differences in the refractive index [28,42]. The near-infrared wavelength is safe as it is neither absorbed by cellular DNA nor generates free radicals, enabling the creation of micrometer-accurate structures [44,45]. Among 3D methods, this approach is considered the best for cell viability (often >80% post-printing, with subsequent proliferation) [43]; however, it is highly expensive and time-consuming [13], and the risk of cell desiccation due to small printing volumes is not negligible. Unlike Laser Bioprinting, the related technique Laser-Induced Forward Transfer (LIFT), despite its high precision, is of low interest for TE due to the high probability of cellular DNA damage [28,42]. Stereolithography (SLA) is also a nozzle-free method, preventing shear stress issues. It relies on the photopolymerization of bioink (exposed to UV or visible light) to build the scaffold layer-by-layer [28,42]. SLA can achieve extremely high resolution (down to a single cell/droplet) and uses high cell densities (up to 10^8^ cells/mL). Its limitations in TE stem from the long overall printing time and the necessity of using bioinks that are both photo-crosslinkable and biocompatible [13].

#### Cell Damage in Laser-Assisted Bioprinting

**Figure 3:**
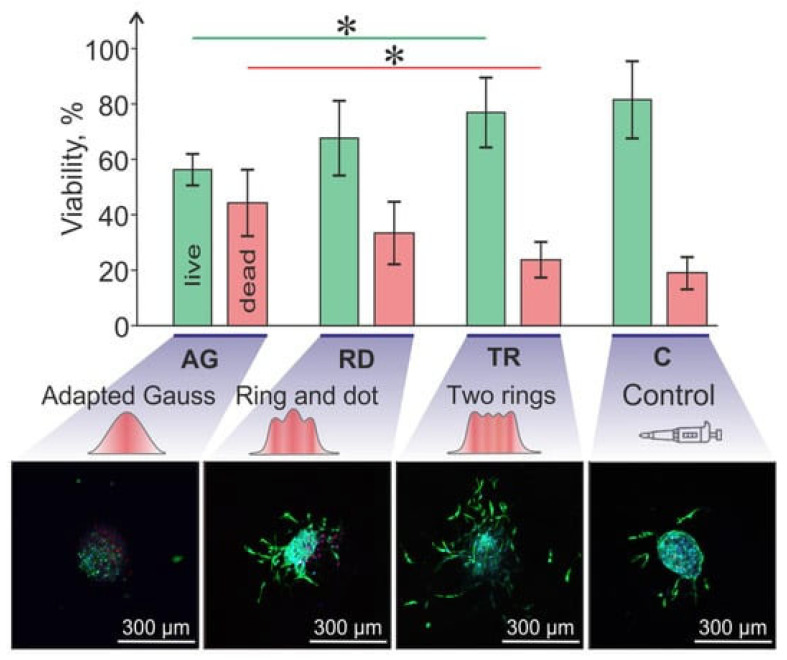
Viability of cell spheroids after laser-induced forward transfer bioprinting, depending on the laser spot shape, compared to a control group of spheroids transferred by means of micropipette. The percent of live/dead cell in spheroid is significantly different with *p*-value < 0.05 (*). Reprinted from [50] and licensed under the Creative Commons Attribution 4.0 International license.

In laser-assisted bioprinting, there are two major causes of cell damage: shear stresses during bioink acceleration (jetting) or deceleration (landing), and the stresses imposed by the laser beam [13]. Shear stress magnitude is determined by bioink viscosity and shear rate [46]. High acceleration during jetting causes significant shear stress, inducing cell membrane damage and DNA breakage. In this context, shear-thinning bioinks can mitigate this damage by reducing viscosity under pressure, which lessens jet acceleration and cell impact forces [28]. The UV laser beam itself can induce enzyme inactivation, protein denaturation, and DNA damage. Also in this case, higher cell concentration increases shear stress. Nonetheless, the overall cell viability associated with laser-assisted bioprinting can be considered high and the technique can be classified as cell-viable [13,47,48]. Mitigation efforts include using visible light for polymerization (though resolution decreases) [49] and incorporating an intermediate quartz-based laser-absorbing layer to protect cells [28]. Moreover, it has been shown that the use of laser spots with different non-Gaussian intensity distribution profile and tunable laser spot size can regulate viability (Figure 3) [50].

### 2.3. Extrusion-Based Bioprinting

Extrusion-based bioprinting is probably the most exploited bioprinting technique used for TE applications [51,52]. It is a rapid prototyping technique able to reproduce even complex structures with high fidelity [24]. Extrusion involves the deposition of biomaterials or bioink through a nozzle-printhead and is categorized as either mechanical or pneumatic printing, based on the forces applied [28]. The first method requires viscous bioinks and converts the rotations of the motor into linear pulses to push the bioink toward the nozzle, while the latter method employs air pressure for extrusion. Common bioink materials in extrusion 3D bioprinting are alginate, gellan gum, agarose, gelatin, collagen, and decellularized extracellular matrix (ECM) [28,53,54]. In particular, shear-thinning materials (e.g., gelatin, silk-fibroin, polyethylene glycol) are the gold standard for pneumatic extrusion.

Extrusion-based techniques are widely applied because of their simplicity, affordability, and relatively good predictability [30]. They allow the handling of a wider range of biomaterials, including also high viscosity materials.

Moreover, higher cell densities can also be used, allowing for wider experimental conditions to be investigated. Despite these advantages, extrusion typically leads to a high rate of disruption of the cellular structure due to high pressure and significant shear stresses at the nozzle outlet [24,30,55]. Regulating mechanical stress is crucial for controlling cellular phenomena like growth, proliferation, and differentiation (mechanotransduction) [56,57,58,59,60]. For example, matrix stiffness, regulated by the hydrogel bioink, acts as a physical cue for cell differentiation [60]. In fact, bioprinted mesenchymal cells showed increased Yes-Associated Protein (YAP) nuclear localization and subsequent differentiation when printed in stiff hydrogels [60]. In addition to matrix stiffness, the dynamic forces imparted during bioprinting extrusion can be exploited. In the work of Heo et al. [61], comb-assisted bioprinting was used to fabricate gingival tissue. The shear forces generated during the extrusion induce mechanotransduction, activating transcriptional regulators pathways and promoting cell alignment. Crucially, this printing-induced mechanotransduction has sustained effects after extrusion, leading to enhanced cell growth, improved cytoskeletal organization, and superior extracellular matrix production over time. Similarly, the MyoColl fibrillar hydrogel platform [62] was optimized for 3D bioprinting by controlling its shear-thinning properties. Its fibrillar architecture and mechanical characteristics effectively induce cellular mechanotransduction in 3D cultures. This mechanical stimulation drives myoblast differentiation, resulting in the formation of myofilaments within the bioprinted constructs, demonstrating a sustained, long-term effect on tissue organization and maturation.

#### 2.3.1. Cell Damage in Extrusion-Based Bioprinting

**Figure 4:**
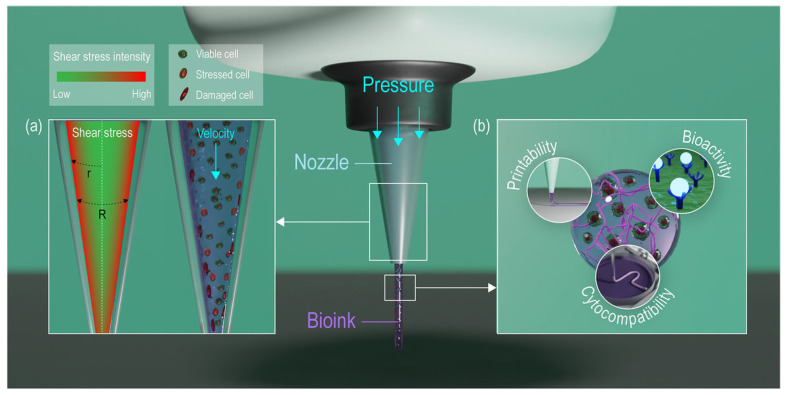
Illustrative images of extrusion-based bioprinting representing: (**a**) the shear stress intensity within the bioink through the nozzle and the consequent cell response; (**b**) the key properties required for an ideal bioink (e.g., printability, biocompatibility, and mechanical stability). This image is reprinted from [64] and is licensed under the Creative Commons Attribution 4.0 International license.

During extrusion-based bioprinting, pressurized bioink moves from the large syringe body (where fluid effects are negligible) into the narrow needle/nozzle, where cells experience significant velocity gradients and stresses based on their radial position (Figure 4). The maximum shear stress is near the nozzle walls, causing cells to deform.

As shear stress increases, cell damage increases exponentially, notwithstanding that cell response to extrusion also depends on the type of cells [56]. Several parameters affect cell viability, including bioink dispensing pressure, nozzle geometry, printing speed, and deposition velocity.

The dispensing pressure is reported to be the dominant factor causing cell damage compared to the nozzle diameter. Indeed, increasing the nozzle pressure leads to a parabolic rise in shear stress and a parabolic decline in cell viability [28]. Chang et al. [63] confirmed that mechanical damage increases the pressure and suggested that a significant portion of cell recovery occurs within the first 24 h post-fabrication.

Shear stresses occurring during extrusion bioprinting can be described by a power law function for non-Newtonian fluids that correlates apparent shear rate and shear stress. Nair et al. [65] reported an empirical model to predict the amount of live, injured and dead cells after bioink extrusion as a function of dispensing pressure and nozzle-tip diameter (Table 1).

Nozzle diameter and viscosity also affect the shear stress that can be generated in the nozzle itself. Moreover, conical needles are generally preferred to cylindrical needles because they require lower pressure to obtain the same fluid velocity leading to a lower cell damage rate [28,66]. Li et al. [67] simulated cell deformation and movement in conical needles. While cells showed a round-shaped maintenance at the nozzle inlet, they deformed as long as they moved toward the nozzle exit. The strain energy density (SED) of the cell membrane was found to increase as the cells moved toward the exit. In the center of the nozzle, the velocity gradient was small compared to that at the walls, leading to a lower cell membrane deformation and SED. When considering the impact of printing speed and deposition velocity on cell viability, it is reported that the higher the pressure applied during the extrusion process, the higher the printing speed and the lower the cell viability. Shear rate at the nozzle wall is directly proportional to the deposition velocity, while it is inversely proportional to the nozzle radius [28,65]. A comparison of cell viability percentage across the different bioprinting techniques, i.e., inkjet-, laser- and extrusion-based bioprinting, is reported in Table 2.

**Table 1:** Effect of nozzle diameters on cell viability using extrusion-based bioprinting.

Bioink	Pressure (kPa)	Nozzle Diameter (μm)	Cell Viability (%)	Reference
0.5–1.5% *w*/*v* alginate, mouse fibroblasts	50 ÷ 150	150 ÷ 300	76 ÷ 96	[34]
0.2% *w*/*v* alginate, Schwann cells and myoblasts	100 ÷ 330	100 ÷ 330	75 ÷ 100	[68]
5–20% *w*/*v* gelatin methacrylamide, HepG2 cells	0 ÷ 500	150 ÷ 200	50 ÷ 100	[66]
2% *w*/*v* alginate and 8% *w*/*v* gelatine, aortic valve interstitial cells	10 ÷ 200	500 ÷ 700	>50	[69]
(5% *w*/*v*) Alginate—(0.6% *w*/*v*) Collagen and 5% *w*/*v* gelatine, human umbilical vein endothelial cells and human dermal fibroblasts	100 ÷ 900	900 ÷ 1900	>70	[70]
3% *w*/*v* gelatine methacryloyl (GelMA) and 3–4% *w*/*v* alginate, mouse fibroblasts	80.5 and 129.1	610	>66	[71]
CELLINK ink (composition/concentration not available), murine myoblasts	35 and 80	200 ÷ 330	77 ÷ 82.1	[72]
2–4–6% *w*/*v* alginate, bovine cartilage progenitor cells	35 ÷ 138	230 ÷ 330 (inner) 1190 (outer)	>60	[73]

#### 2.3.2. Hand-Held Deposition

Hand-held bioprinting is a highly portable, extrusion-based modality designed primarily for in situ bioprinting, enabling the direct, intraoperative repair of tissue defects [74]. The core advantage of this technique is the operator-controlled deposition, which offers superior flexibility and real-time adaptability to irregular and dynamic anatomical geometries—a major constraint for fixed robotic systems [75]. For example, Hakimi et al. [76] present a handheld skin printer enabling the in situ formation of biomaterial and tissue sheets directly onto wound surfaces. The compact extrusion-based device uses a microfluidic cartridge to deposit homogeneous or architected sheets. In this context, post-bioprinting viability, measured on encapsulated human dermal fibroblasts, consistently exceeded 90%.

The hand-held process requires rapid crosslinking (e.g., UV light or chemical activation) to stabilize the bioink immediately after it is dispensed onto the target site [77]. This approach is cost-effective and eliminates the logistical challenges associated with ex vivo construct fabrication and transfer [78]. However, its manual operation leads to significant technical drawbacks: low precision and poor repeatability. The fidelity of the printed structure is highly dependent on the surgeon’s dexterity, which complicates standardization necessary for clinical application [75]. Furthermore, controlling parameters precisely to minimize shear stress and ensure cell viability remains a challenge without automated feedback mechanisms. Despite these limitations, the ability to enable on-site personalized tissue repair makes handheld bioprinting an important advancement in regenerative medicine.

#### 2.3.3. Cell Damage in Hand-Held Deposition

The critical biological challenge specific to hand-held extrusion systems stems from the variable mechanical forces applied during manual operation, which translate directly into uncontrolled shear stress exerted on the cells [65]. Unlike automated bioprinters with calibrated pressure regulators, hand-held devices lack precise, closed-loop control over the extrusion force and speed, making the process highly dependent on the operator’s performance over time. This variability in deposition directly risks cell membrane disruption and apoptosis. Quantitative studies focusing on optimized hand-held printing protocols, such as those employing the Biopen system with gelatin-methacrylamide (GelMa)/hyaluronic acid-methacrylate (HAMa) bioinks, have demonstrated promising results in controlled settings. Specifically, human adipose stem cells (hADSCs) successfully maintained a high viability of over 97% one week after being printed with the Biopen [77]. However, this high rate is achieved under stringent conditions, including the use of shear-thinning bioinks designed to reduce stress within the nozzle. In contrast, general extrusion research shows that cell viability is significantly preserved when low nozzle shear stress (below 5 kPa) is maintained, but it can drastically fall to 76% or lower when the stress exceeds 10 kPa [77,79].

Therefore, the inherent lack of standardized force during manual extrusion represents a significant, long-term threat to functional tissue regeneration, necessitating the development of intelligent bioinks that provide robust buffering against uncontrolled operator variability. This challenge is being addressed by externally controlled systems. For example, the handheld arthroscopic device developed by Guarnera et al. [80] demonstrated that maintaining a controlled pneumatic pressure of 5, 6, or 30 kPa resulted in a high chondrocyte viability after 7 days.

**Table 2:** Comparison of cell viability percentage across the different bioprinting techniques.

Bioprinting Method	Bioink	Cell Viability	Reference
Inkjet-based	1% *w*/*v* sodium alginate, mouse fibroblasts	>90% after 24 h	[81]
	1–2% *w*/*v* sodium alginate, mouse fibroblasts	>82% after 72 h	[82]
	10–20 *w*/*v* poly(ethylene glycol) dimethacrylate, human chondrocytes	>85% after 24 h	[83]
Laser-based	(Concentration not determined) biopaper, human endothelial cells	>90% after 30 min	[84]
	4% *w*/*v* alginate and murine fibroblasts	>90%	[85]
	15% hyaluronic acid solution (1% *w*/*v*), 85% E8 medium, human induced pluripotent stem cells	>90%	[86]
Extrusion-based	1% *w*/*v* alginate sulfate—1.36% nanocellulose, bovine chondrocytes	>81% after 24 h	[87]
	Methacrylamide-modified gelatin (Gel-MA), methacrylated κ-carrageenan (Car-MA) (final concentrations not determined), adipose tissue-derived stem cells	>90% after 14 days	[88]
	2–4% *w*/*v* sodium alginate, mouse fibroblasts and smooth muscle cells	>90% after 7 days	[89]

## 3. Cell Mechanotransduction Mechanism: Cell Response to Mechanical Stress

**Figure 5:**
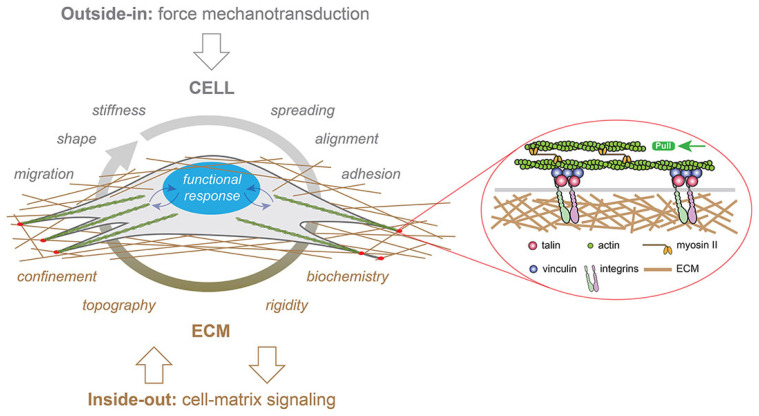
Representation of mechanotransduction signaling in a cell cultured within a three-dimensional matrix. External forces applied to cells modulate their migration, morphology (shape), stiffness, spreading, alignment, and adhesion behaviors. Concurrently, the ECM provides multiple cues to the cells, including confinement, topography, rigidity, and biochemistry. Mechanical signals (represented by curved blue arrows) are converted into biological responses by the nucleus, depicted in blue. This image is reproduced from [91] and licensed under the Creative Commons Attribution 4.0 International license.

Mechanotransduction is the cellular process of local sensing and responding to localized mechanical forces and the physical microenvironment [90]. This critical response is characterized by a three-step pathway [56]: it begins with mechanosensing via extracellular proteins (e.g., integrins and cadherins), followed by signal transduction process (mechanotransduction) through the cytoskeleton (involving talin and vinculin), and culminates in nuclear communication via nesprins, ultimately leading to a cellular response such as growth, differentiation, or morphological change (Figure 5) [56].

Mechanotransduction is essential for TE as mechanical cues are fundamental to tissue development and effectively guide stem cell differentiation based on matrix stiffness [64,65,66]. In extrusion-based bioprinting process, the primary mechanical stimulus is applied to cells embedded in the bioink during the extrusion phase, then the resulting cascade of cellular events can evolve over time after extrusion, leading to sustained biological outcomes. In this context, mechanical forces must be tightly controlled, as process-associated stresses (e.g., during bioprinting) can exceed desired magnitudes, inducing cell membrane damage and reducing viability [13,56,58].

Computational models can be an important tool to predict cell response to printing process parameters. In particular, CFD modeling is employed to predict cellular response to shear stress. This approach can be used for the optimization of parameters, including nozzle geometry (e.g., 20–30° angles [55]) and material selection, with studies highlighting the importance of specialized inner coatings like Ethylene Diamine Tetra-acetic Acid (EDTA) [92] or the use of glass nozzles [93,94,95] to modulate friction and minimize detrimental stress exposure.

## 4. Simulation of the Printing Process Through Computational Fluid Dynamics

CFD is a branch of fluid dynamics that uses a combination of numerical procedures and computational software to solve complex fluid-related problems. In the bioprinting field, CFD is a highly useful tool that allows testing the effects of specific printing parameters without the need to perform several experiments with different protocols, limiting the experimental tests as final validation. As described by Blanco et al. [55], the simulation of material flow in the bioprinting process facilitates understanding the interrelationships between nozzle geometry, printing parameters, material properties, and mechanical forces during extrusion, allowing for the prediction of mechanical stress exerted on extruded bioink. CFD can also help in optimizing the printing process and developing novel bioink formulations. Printing parameters, as printing speed, nozzle geometry, dispensing pressure, and rheological properties of the bioink, can be modeled and set according to the results of numerical simulation [96]. As already discussed, extrusion bioprinting can have the highest burden on cell viability; for this reason, the following sections are focused on currently available CFD studies for solving cell extrusion-related problems.

### Predicting Cell-Associated Stress During Extrusion Bioprinting

Current studies on extrusion bioprinting generally focus solely on bioink modification, processing methods, and printing equipment, mostly neglecting the effect of extrusion on cell viability [97]. In fact, for the last aspect, CFD analysis can provide a tool to understand the correlation between bioprinting process parameters and cell damage, allowing evaluation of stresses and flow distribution in the printing needles. According to several studies [63,98,99,100], the degree of cell damage depends on the type of cells that are printed: fibroblasts undergo 40% of damage, hepatocytes are instead less affected (2% of them are effectively damaged), while 6% of chondrocytes can be injured. Cell viability is correlated to different parameters, both linked to cell manipulation techniques and printing process parameters (system pressure, needle diameter, and needle length). Clearly, cell damage is an issue that needs to be assessed to improve the biological outcomes of bioprinted scaffolds. A way to describe extrusion cell damage typically involves the introduction of mathematical models linking cell damage with printing parameters (e.g., shear stress and printing time). A simple law was proposed to model cell damage [13,56]:

(1) $$I(\%)=C\tau^{a}t^{b}$$

where *I*(%) represents the percentage of cells being damaged, *τ* the shear stress to which cells are exposed, and *t* the time of effective exposure to printing stresses. The scalar *C*, *a*, and *b* are parameters that vary according to the type of cells. This predictive law has some limitations, since it neglects the damage probability distribution and a possible correlation factor between stress and exposure time. Moreover, this equation cannot be used with high values of stress and exposure time, which would result in predicted values of *I* exceeding 100% of damaged cells [56,101]. In addition, the spatial distribution of stress is not considered, even though it is known that the shear stress increases with the needle radius, is null at the needle center, and is maximum at its walls [97]. Li et al. [101] described the effect that hydrostatic and shear stresses have on cells, validating the mathematical model with established experimental protocols. To observe the effect of hydrostatic pressure, cells were introduced into a syringe with a removable cap screwed in place of a needle. A hydrostatic pressure was applied to the syringe, and the effect of the pressure field was evaluated. On the other hand, shear-induced cell damage was settled through a shear-inducing rheometer. The authors also improved the model proposed in Equation (1) introducing a bivariate normal distribution function. In this way, the percentage of cell damage under pressure or shear stress *CD*_pr/sh_ is expressed as a function of stress *s* and exposure time *t*:

(2) $$CD_{pr/sh}(s,t)=\iint_{s,t}f(s,t)dsdt$$

(3) $$f(s,t)=\frac{1}{2\pi SD_{s}SD_{t}\sqrt{1-\rho^{2}}}\exp\left[-\frac{z}{2\left(1-\rho^{2}\right)}\right]$$

(4) $$z={\left(\frac{s-\bar{s}}{SD_{s}}\right)}^{2}-2\rho \cdot \frac{s-\bar{s}}{SD_{s}}\cdot \frac{t-\bar{t}}{SD_{t}}+{\left(\frac{t-\bar{t}}{SD_{t}}\right)}^{2}$$

where *f*(*s*,*t*) represents the probability density function, while $\bar{s}$ and $\bar{t}$ represents stress and time mean values. The scalar *t* is the exposure time, *SD_s_* and *SD_t_* are the standard deviations of *s* and *t*; finally, *ρ* is the correlation coefficient between *s* and *t*. To obtain these parameters, the experiments described above were carried out and cells in suspension were considered to follow non-Newtonian flow behavior. To validate the model, an experimental extrusion process was performed by using sodium alginate including suspended Schwann cells and 3T3 fibroblasts. Cell damage was found to increase with pressure increase and with needle diameter decrease. Shear stress was related to needle length and radial position, with exposure time affecting the same way the stress field. Hydrostatic pressure did not influence cell viability at all [56,101].

Müller et al. [102] reported a three-dimensional model based on the lattice Boltzmann method for the flow profile of shear-thinning fluids at the exit of a printing nozzle. This numerical simulation was proposed to evaluate the cell/stress and cell/strain correlations for centered and off-centered cells [54,102]. Cells were modeled through a hyperelastic constitutive model involving a coupled Eulerian–Lagrangian approach to simulate interaction phenomena between bioink and cells [102]. The authors also attempted to distinguish between two different modes in which cells are subjected to stress. The first one is related to viscous stresses that are mainly caused by frictional motion of the cell interior (e.g., tank treading), while the second one is connected to the elastic stress caused by shearing and stretching mode deformation modes. If the former stress is determined by cell intrinsic viscosity, the latter is due to cell elastic moduli. Considering that both mathematical and numerical models need a validation step, the authors simulated two different experimental conditions: the flow inside the nozzle and the flow exiting it (transition of the biomaterial flow from bounded to free-standing). While flowing into the center of the nozzle tip, cells assumed a bullet-like deformation; instead, cells flowing off-center deformed into an ellipsoidal shape following a tank-treading motion [102]. Finally, during the free-standing printing, cells were exposed to elongational flow patterns. Nonetheless, radial deformation seems to also interest cells that are flowing out of the center, in that case with predominance of the shear deformation. For those cells that are in the central zone of flowing biomaterial, high magnitude radial stretches occur.

**Figure 6:**
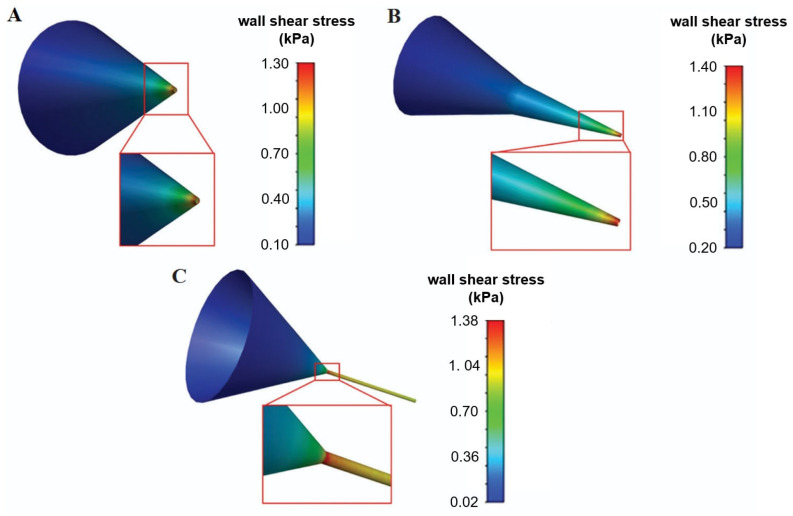
Contours of wall shear stress for the (**A**) tapered conical, (**B**) conical, and (**C**) cylindrical nozzles with an exit diameter of 300 μm and an upstream pressure of 200 kPa. Adapted from [103]. This file is licensed under the Creative Commons Attribution 4.0 International license.

Different nozzle geometries can also impact cell viability and biomaterial printing outcomes (Figure 6).

In this regard, Wei et al. [97] proposed some computational models based on CFD and the use of COMSOL Multiphysics^®^ to describe the effect of different nozzle geometries on the fluid extrusion process. Cylindrical and conical needle models were developed, exploiting the axial symmetry of the systems to be analyzed in order to reduce the computational cost. The extrusion of sodium-alginate bioink through these needles was simulated to estimate steady pressures, flow velocity, and shear stress were investigated. Compared to cylindrical nozzles (at constant inlet pressure and outlet diameter), the steady-state pressure of the bioink in conical needles varied steadily, which was due to dynamic bioink adjustment. Moreover, under constant inlet pressure, the steady-state pressure into the conical nozzle decreased exponentially. Increasing the outlet diameter, the pressure changed more smoothly, and the flow rate at the outlet increased. At low speed flow, the shear stress inside the two nozzles was dominant: the closer the molecules were to the wall, the greater the shear stress. The peak of wall shear stress detected in the conical nozzle was higher than that occurring in the cylindrical one: indeed, the wall shear stress in the cylindrical nozzle was estimated to be about 30 Pa·s, while that in the conical nozzle was about 18 Pa·s [97]. This indicated that the cumulative damage acting on the biomaterial and cells in the cylindrical nozzle was significantly greater than that in the conical nozzle. Therefore, the selection of the conical nozzle may be beneficial to the bio-friendliness of the bioprinting process [97]. Chand et al. [103] compared the effect of different printing parameters on the maximum wall shear stress in printing nozzles, considered as the most critical factor influencing cell viability. Three nozzle geometries (conical, tapered conical, and cylindrical) and different nozzle diameters (0.1 mm–0.5 mm) were investigated. Steady-state flow was considered, and a non-Newtonian power law was used to model the bioink volume. The authors found that, even if the maximum wall shear stress has the lowest values in cylindrical nozzles, it lasts longer and for a longer nozzle portion compared to other geometries, thus leading to lower cell viability.

Li et al. [67] provided a mathematical model to reproduce the cell damage due to mechanical loading during bioprinting. They analyzed the effect of different printing flow rates on the degree of cell damage. Different solutions were also proposed in order to reduce the shear exerted on cells, as increasing the nozzle diameter and decreasing the bioink viscosity [65,73]. Ning et al. [68] described a method to evaluate the effect that both shear and extensional stresses have on cell damage due to bioprinting. In this study, a rheological experimental setup was exploited to evaluate the effect of shear stress on the selected cells (Schwann cells and myoblasts), while the damage related to extensional stress was obtained as the difference between the damage induced by the overall process and the one correlated to shear. Limited cell damage occurred when low printing pressure was exploited (high shear stress), while high cell damage happened when higher pressure was reached and high extensional stress occurred [68]. Emmermarcher et al. [104] applied numerical modeling to model the fluid flowing through the printing head during the extrusion process. Mechanical stresses, pressure gradient, and flow rate during printing were evaluated. CFD analysis allowed the introduction of dimensionless parameters in order to describe the flow independently of the needle diameter and pressures. To this purpose, a bioink-related nomogram was introduced, and a fast and easy method for the identification of printing parameters was established [55,96,104,105]. The potential of the CFD technique has also been increased and verified experimentally through fluorescence imaging analysis. In fact, while CFD analysis allows for simulating what happens during extrusion, imaging techniques could support in validating mathematical analysis [106].

Poologasundarampillai et al. [106] investigated 3D printing techniques associated with cell damage through a real-time imaging protocol. Light-sheet fluorescence microscopy was applied to investigate the real-time flow of bioink through a capillary, mimicking the typical bioprinting conditions. Different biomaterials were studied as bioink, while cell tracking allowed for the determination of their flow profile. As reported by the authors, the three-dimensional continuous imaging of extrusion flow gave insight into the dynamics of bioink flow and cell movement during the process, helping in coupling ex situ rheological studies and mathematical models [106]. At the center of the capillary, cells suspended in sol–gel based biomaterials exhibited the highest flow velocities and a parabolic velocity profile. In this region, cells appeared elongated, while cells located at the capillary walls had low velocities and a more defined shape. Some cells rolled on the surface of the capillary, while those near the wall, but not in direct contact, spun. Through real-time imaging techniques, the authors deduced the exact flow behavior of the biomaterial tested, allowing for discrimination the more accurate fluid model for each biomaterial [106].

## 5. Methods to Enhance Printing and Post-Printing Cell Viability

As discussed in the previous sections, cell viability is affected by mechanical stress and process parameters during printing, and by bioink chemical composition or scaffold handling post-printing. A core strategy to improve outcomes involves the optimization of the bioink properties, ensuring key requirements such as biocompatibility, mechanical integrity, favorable crosslinking, printability, and appropriate rheology [6,107,108].

Biomaterial selection involves a trade-off: natural polymers (e.g., alginate, collagen, fibrin, gelatin) boast high biocompatibility, but often lack mechanical strength, while synthetic materials (e.g., poly(e-caprolactone), PEG-based polymers, Pluronic^®^) offer tunable physical properties despite being characterized by lower levels of biocompatibility. Both material types rely on polymer chains to provide the high-water retention and hydration essential for cellular functions [109,110]. Natural polymers commonly utilized include alginate, chitosan, agarose, hyaluronic acid, collagen, fibrin, gelatin, and decellularized ECM [111,112]. Composite biomaterials are gaining attention for combining the benefits of both classes [111,112,113]. Among all types of biomaterials, hydrogels are particularly favored for their ability to reproduce a cell-friendly, hydrated matrix with adjustable chemistry and porosity, allowing for nutrient perfusion and cell migration [112,114].

Critically, poor rheological properties, such as uncontrolled viscosity, lead to cell sedimentation, nozzle clogging, inhomogeneous distribution, and expose cells to damaging loads. Therefore, mitigation efforts involve refining printing parameters and adjusting the chemical and mechanical properties of the biomaterials. Specific techniques employed to enhance viability include the development of interpenetrating and reversible hydrogels, the encapsulation of cells within stress-shielding microparticles, and the implementation of pre-printing cell conditioning approaches.

### 5.1. Interpenetrating, Thixotropic, and Reversibly Crosslinked Hydrogels

Chemical modification of the bioink materials is a primary strategy to improve cell viability within 3D-printed scaffolds. Dubbin et al. [114] introduced the Mixing-Induced Two Component Hydrogel (MITCH) [115,116], a new gel-phase bioink with dual-stage crosslinking, designed to enhance cell distribution homogeneity and mechanical protection of the cell membrane during printing. The MITCH, formed via a 1:1 combination of modified alginate and an engineered peptide, initially exhibited a storage modulus of approximately 20 Pa, corresponding to a suitable stiffness to shield cells from mechanical damage during bioprinting.

Post-printing, the physical crosslinking was reinforced by divalent cations, increasing the average storage modulus to 4 kPa [114,117,118]. This approach resulted in high residual viability for encapsulated fibroblasts and pluripotent stem cells, maintaining cell homogeneity even after 1 h of printing [114].

In addition to chemical modification, the bioink viscosity is also a critical parameter: low viscosity is favorable to extrusion, but it can lead to post-printing structure collapse; differently, high viscosity can induce significant shear stress during extrusion, leading to reported cell survival rates of only 40–80%.

To address this, thixotropic bioinks, such as the one developed by Cui et al. [119] (gelatin, tannic acid and, sodium alginate pre-crosslinked with calcium ions, Ca^2+^) require lower extrusion forces, thus preserving cell viability [52,120,121,122]. Similarly, microencapsulation techniques demonstrated significant quantitative gains. Wang et al. [123] microencapsulated islet cells in type I diabetes mellitus treatment into an interpenetrating network of alginate and ECM hydrogel. The result was a sevenfold increase in cell growth over one week in vitro and a robust glucose-stimulated insulin response, highlighting the potent increase in cell function compared to traditional therapies.

### 5.2. Three-Dimensionally Printed Hydrogel Curing Though Calcium Ion Deposition

Post-printing curing of 3D-printed hydrogels is a critical step known to significantly affect cell viability and biological performance in bioprinting. Fischer et al. [124] capitalized on the well-established effect of Ca^2+^ ions in cell membrane repair, to achieve simultaneous curing and cell viability enhancement. Calcium is fundamental in cell signaling pathways, regulating proliferation and gene expression as a key intracellular messenger. Crucially, the passive entrance of Ca^2+^ through cell membrane leakages (often induced during the bioprinting process) was reported to increase plasma membrane resealing and facilitate cytoskeleton remodeling [124,125,126]. Considering this, they hypothesized that calcium supplementation into the bioink material would mitigate shear stress-related damage during extrusion, thus improving cell viability. Results obtained from cell extrusion in alginate bioink confirmed that the supplementation of a physiological concentration of Ca^2+^ led to an enhanced overall cell survival. Moreover, a quantitative comparison was established by subjecting different cell types—characterized by varying membrane stiffness—to identical printing conditions. Soft cells, which exhibit greater deformation than stiff cells under constant shear stress, demonstrated the most pronounced benefit from calcium addition due to an improved resealing of damaged plasma membranes [127,128,129,130].

### 5.3. Hybrid Scaffold Production Inkjet Bioprinting

**Figure 7:**
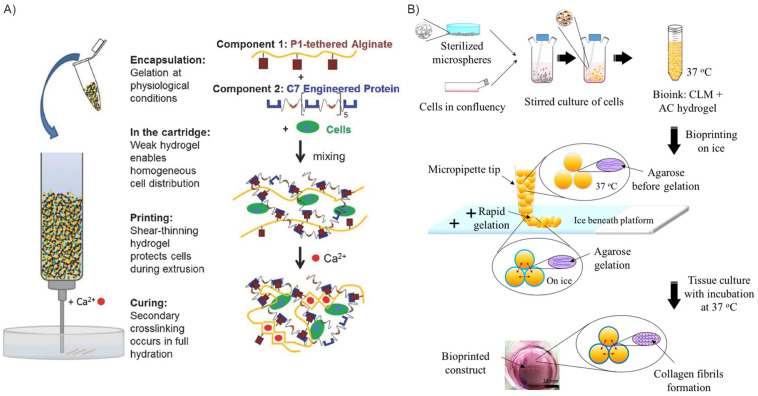
Examples of different approaches to enhance cell viability. (**A**) Alginate calcium-cured bioprinted construct is intended for a dual stage crosslinking: noncovalent binding between two complementary peptides to form a weak gel upon mixing of the two polymer components; ionic crosslinking occurs between calcium ions in solution and the alginate backbone of the component. Reprinted from [114] with kind permission from John Wiley and Sons. Copyright 2016 WILEY-VCH Verlag GmbH & Co., KGaA, Weinheim, Germany. (**B**) PLGA microspheres are used as cell homing and seeding structures. The embedded microspheres are intended to be encapsulated into a thermoresponsive hydrogel to can be further bioprinted. Reprinted from [132] and licensed under the Creative Commons Attribution 4.0 International license.

Hybrid bioprinting methods are increasingly utilized, exploiting natural materials for biocompatibility and microenvironment support, and synthetic materials to confer desired mechanical properties and biodegradability. In this regard, Kim et al. [131] developed a novel bioprinting method to produce a 3D cell-printed scaffold composed of PCL/poly(lactic-co-glycolic acid) (PLGA) and tricalcium phosphate (TCP). PCL was introduced to act as a protective thermal layer against damage induced by the PLGA dispensing temperature (140 °C). PCL layers were strategically printed adjacent to where cells would be susceptible to thermal exposure during the PLGA fabrication process. The study confirmed that PCL layers, when positioned between the thermal damage-inducing areas, successfully protected the cells in the printed construct from thermal degradation. The overall process is briefly described and schematically represented in Figure 7A.

### 5.4. Cell Encapsulation into Microparticles and Microbeads Inkjet Bioprinting

While hybrid material strategies can offer thermal protection [131], an alternative solution to reduce cell damage is the use of cell-homing microcompartments (e.g., microgels, microspheres, droplets). These microscale protective niches shield embedded cells from excessive shear stress, simultaneously supporting post-printing viability and function by providing physical protection and localized biochemical cues.

Tan et al. [132] demonstrated this by fabricating cell-laden PLGA porous microspheres to be included in a collagen-agarose hydrogel. These microspheres served as pre-printing cell-seeding and expansion sites, while the hydrogel acted as a rapid-gelling delivery vehicle. This printing method leads to cell damage reduction, as the steric size of the particles reduces the effective shear stress experienced by the encapsulated cells during bioprinting. Similarly, Rousselle et al. [133] developed a bioink incorporating poly(D,L-lactic-co-glycolic acid) solid microspheres with methacrylate collagen and hyaluronic acid. Cells were allowed to colonize and proliferate within the microspheres before being co-extruded with the bioink. With this approach, encouraging quantitative results were obtained: cell survival increased by 10% compared to both hand deposition and standard extrusion bioprinting performed without the exploitation of microscaffolds (Figure 7B).

### 5.5. Shear Stress Preconditioning

Pre-print cell training represents a unique strategy to improve cell viability by focusing on cell behavior rather than biomaterial modifications [72]. In particular, the idea resides in the concept of cell preconditioning [134,135,136,137], a common procedure in stem cell application, which can enhance the therapeutic and regenerative impact of cells in tissue engineering [138].

Two main strategies are typically exploited for this goal: the first is based on the amplification of specific positive cellular signals, the second is based on the exertion of injury-like stresses. Boularaoui et al. [72] applied the latter by investigating the possibility of preconditioning cells to tolerate stress induced by printing. Using a custom-made device, they performed shear stress preconditioning on C2C12 murine myoblasts and then evaluated post-printing cell viability. They observed that pre-exposing the cells to a controlled shear stress of about 1 Pa prior to bioprinting could significantly enhance their viability following the printing process. In particular, preconditioned cells exhibited a 7.8% higher viability than non-conditioned cells when printed through a nozzle, and a 6.6% higher viability when printed through a needle.

## 6. Discussion and Conclusions

The literature confirms that keeping cells alive during 3D bioprinting is the main challenge related to this technique, especially due to the non-physiological loading conditions and to the excessive shear stress exerted on cells during the process. While extrusion bioprinting is widely used and versatile, it exposes cells to stress levels that severely reduce survival. The most crucial development is the shift from simple testing to using CFD modeling. CFD allows for quantitatively estimating the stress state during bioprinting under specific conditions and therefore to predict cell damage, confirming that some cell types are naturally tougher than others under the same applied stress, which means that printing recipes must be customized for different cells. CFD also directly improved the equipment itself, showing that the use of conical nozzles (instead of standard ones) significantly reduces shear forces and thus offers a simple way to protect cells. Guarnera et al. [80], for example, employed CFD modeling to predict shear stress on the 1.4 mm inner tube wall of the extrusion device in the worst-case (90° bent tip) scenario, verifying that the generated stresses were compatible with cell safety. The CFD results, validated with a 5% maximum error, confirmed that shear stress levels remained well below the critical 5 kPa safety threshold, guaranteeing high cell viability during extrusion.

Based on these threats analyzed, three effective strategies have been developed to increase cell survival:

1. Design of protective bioinks: the goal is to create a printing material that protects cells against mechanical stress. Innovations like the MITCH [114] use a clever two-step hardening process: the material stays soft during printing (to protect the cells) and then quickly locks into a strong structure afterward. This separates the need for easy printing from the need for a strong final scaffold. Simple chemical changes also play a supportive role; for example, the addition of specific ions promotes the repair of damaged cell membranes, which is especially helpful for the most fragile cells after printing [124].

2. Physical shields and hybrid printing: this strategy involves building physical protection. For heat problems, hybrid printing works by placing heat-resistant materials near hot parts. More importantly, the encapsulation of cells in microcompartments (tiny protective spheres) acts like a cushion against applied stress. Preliminary literature studies [123,139] clearly show that these shields strongly increase the final percentage of surviving cells by absorbing external forces during extrusion.

3. Cell training (pre-conditioning): this is a biological solution focused on strengthening the cells themselves. By exposing cells to controlled, mild stress before they are printed, the cells can be trained to better handle the extreme forces they face in the printing nozzle. This method successfully improves the cells’ ability to survive the process, confirming that we can use the cells’ natural defenses to our advantage.

In summary, the future of bioprinting relies on combining these targeted strategies. Optimization of delivery and printing parameters, development of smarter bioink systems that protect cells during delivery, and incorporation of cellular pre-conditioning are essential to produce engineered tissues that are both structurally sound and fully viable and functional.

## Data Availability

No new data were created or analyzed in this study. Data sharing is not applicable to this article.

## Abbreviations

The following abbreviations are used in this manuscript:

3D	Three-dimensional
UV	Ultraviolet
CFD	Computational fluid dynamics
LIFT	Laser-induced forward transfer
hMSCs	Human mesenchymal stem cells
HUVEC	Human umbilical-vein endothelial cells
HUVSMC	Human umbilical-vein smooth muscle cells
YAP	Yes-associated protein
EDTA	Ethylene Diamine Tetra-acetic Acid
PCL	Polycaprolactone
PLGA	Poly(lactic-co-glycolic acid)
MITCH	Mixing-induced two component hydrogels
TCP	Tricalcium phosphate
ECM	Extracellular matrix
